# Retrospective quality of life study in patients with retroperitoneal sarcoma in an Asian population

**DOI:** 10.1186/s12955-020-01491-0

**Published:** 2020-08-06

**Authors:** Hui Jun Lim, Chin-Ann Johnny Ong, Thakshayeni Skanthakumar, Lisa Yuen Hong Mak, Seettha Devi Wasudevan, Joey Wee-Shan Tan, Claramae Shulyn Chia, Grace Hwei Ching Tan, Melissa Ching Ching Teo

**Affiliations:** 1 Department of Sarcoma, Peritoneal and Rare Tumours (SPRinT), Division of Surgery and Surgical Oncology, National Cancer Centre Singapore, 11 Hospital Crescent, Singapore, 169610 Singapore; 2 Duke-NUS Medical School, 8 College Road, Singapore, S169857 Singapore; 3 Laboratory of Applied Human Genetics, Division of Medical Sciences, National Cancer Centre Singapore, 11 Hospital Crescent, Singapore, S169610 Singapore; 4 Institute of Molecular and Cell Biology, A*STAR Research Entities, 61 Biopolis Drive, Singapore, S138673 Singapore

**Keywords:** Retroperitoneal sarcoma, Quality of life, Surgical oncology

## Abstract

**Background:**

Retroperitoneal sarcoma represents 15% of sarcomas. The mainstay of treatment is surgery where a majority of patients require multi-visceral resections that may significantly impact their quality of life (QOL) following surgery. Studies in other cancers have shown that QOL may not be significantly impacted after radical or extensive surgery. However, there are limited studies examining the QOL specifically in patients with retroperitoneal sarcoma. In this pilot study, we retrospectively evaluated the QOL of patients with retroperitoneal sarcoma.

**Methods:**

32 out of 90 patients who underwent surgical intervention for retroperitoneal sarcoma in National Cancer Centre Singapore from January 1999 to August 2018 who were alive and on follow-up were included in this study. EORTC-QLQ-C30 was administered to the patients.

**Results:**

The median age of our patients was 59 years (range, 35–84), and median time from surgery to the implementation of questionnaire was 2.5 years (range, 0.05–9.6). Younger patients had significantly better differences in global health, physical and role functioning scores as compared to older individuals. Female patients reported higher global health, physical, emotional and social functioning scores than males. Patients who were more than 2 years post-surgery exhibited better QOL scores as compared to those who had more recent surgery. Our patients had comparable global health and functioning scores compared to a reference group of outpatient cancer patients at our institution.

**Conclusions:**

Our pilot study investigating the QOL of patients with retroperitoneal sarcoma has shown that patients need to be followed up for at least 2 years following surgery to evaluate their QOL. In general, they achieved better functioning scores when compared with other cancer patients. These findings support the need for larger-scale prospective studies to further evaluate the QOL of these patients.

## Background

Sarcomas are a rare and heterogeneous group of malignant tumours of mesenchymal origin that comprise less than 1% of all adult malignancies [1]. They are a histologically heterogeneous group of malignant tumours with highly variable characteristics and clinical outcomes. Additionally, sarcomas can originate in any part of the body, such as the extremities, trunk wall, retroperitoneum and head and neck [2, 3].

Retroperitoneal sarcomas account for approximately 15% of soft sarcomas [2, 3]. Notably, there are various subtypes of retroperitoneal sarcomas, including liposarcoma, leiomyosarcoma and synovial sarcoma, which account for 75% of all cases [2]. Due to the site and pattern of growth in the retroperitoneum, these tumours often grow to a substantial size before the patient’s non-specific complaints are evaluated or even before an abdominal mass is noted on physical examination. As such, retroperitoneal sarcomas are often large and anatomically in close proximity to critical normal structures and organs within the abdomen and pelvis [2]. The five-year overall survival rate for patients with retroperitoneal sarcoma is approximately 50% and declines to 20 to 30% at 10 years [3, 4]. Reports indicate that loco-regional recurrences are observed in 40 to 50% of the patients within the first 5 years following surgery [3]. While variation is seen between histological subtypes, retroperitoneal liposarcoma has a predilection for local recurrence where approximately 20% of patients develop distant metastases by 5 years following treatment [4].

The mainstay of treatment for retroperitoneal sarcoma is complete resection with curative intent, which is integral for improved survival [5]. The ability to achieve a complete resection largely depends on the relation of tumour to major vascular structures and whether there is an invasion of adjacent visceral organs [6–8]. Due to the complexities involved, many of these patients who require multi-visceral resections are concerned about the impact of surgery on their quality of life (QOL). Studies in other cancers have shown that QOL dips initially following surgery but returns to baseline in long-term survivors [9, 10]. However, there are limited studies examining the QOL specifically in patients with retroperitoneal sarcoma following surgery, especially in an Asian population. The results of other QOL studies may not be applicable to our local patient population with limited generalisability as cognitions and perceptions about health and illness may vary between cultural settings. Patients of varying backgrounds have been shown to place different values on health outcomes of treatment. For instance, patients of an Asian background may prioritise the needs of their family over their own [10, 11]. In the present pilot study, we aim to retrospectively evaluate the QOL of patients with retroperitoneal sarcoma to understand the trends of various aspects of QOL after surgery, which will provide greater insight into their functional and social status post-operatively.

## Methods

This is a retrospective cross-sectional study. All patients who had retroperitoneal sarcoma in the National Cancer Centre Singapore (NCCS) from 1st January 1999 to 31st August 2018 and underwent curative surgery were included. Participants were identified through past medical and operative records. Those who were living at the time of study commencement and still on active follow-up were eligible. A total of 90 patients underwent curative surgery during this period but 43 had passed away. Of the 47 patients who were still alive at the time of study commencement, 15 were lost to follow-up. Attempts to contact them were unsuccessful. The remaining 32 patients were invited to participate in the study. If the patients had an appointment within a month of the study commencement, they were recruited during their clinic session and the QOL questionnaire was administered during the session by a research assistant. For patients with follow-up appointments more than a month away, they were contacted by a research assistant via telephone. If the patient was agreeable to participate, the questionnaire was either administered over the phone or a meeting was arranged. Informed consent was obtained from all participants. All 32 patients (100%) were agreeable to participate in the study.

The European Organization for the Research and Treatment of Cancer Core Quality of Life Questionnaire (EORTC QLQ-C30) was used to assess their QOL after surgery. The EORTC QLQ-C30 is designed for use with a wide range of cancer patient populations and has been used in multiple studies worldwide [11]. The questionnaire comprises of five functional scales, three symptom scales, six single symptom items and a global health-related QOL score. Nine symptom items in the EORTC QLQ-C30 include a three-item symptom scale measuring fatigue, two symptom scales measuring pain and nausea and vomiting, and six single-item symptom scales measuring dyspnoea, insomnia, appetite loss, constipation, diarrhoea and financial impact. Specifically, patients are assessed to have higher levels of functioning when they scored higher on the functional scales and global health-related QOL score. On the contrary, patients who are experiencing more symptoms at the time of assessment would reveal higher scores on the symptom items. The EORTC manual provides reference values for comparison. However, a third of the data was taken from EORTC studies performed in Western populations. In comparison, a study published in 2004 used the EORTC QLQ-C30 to assess QOL of 396 cancer patients in our local context [11]. These patients were recruited from NCCS during their follow-up appointment and were on active follow-up for their cancer. The questionnaire was administered to these patients while they were in the waiting areas of specialist outpatient clinics, the Ambulatory Treatment Unit or the Therapeutic Radiology Department. As this reference group of patients are more similar to our study cohort in terms of demographic characteristics, we used their scores as the reference value for comparison to gain a more accurate evaluation into the QOL of patients with retroperitoneal sarcoma as compared to other cancer patients. Complications that arose post-operatively were classified according to the Clavien-Dindo Classification. The Clavien-Dino Classification is a useful system to rank a post-operative complication in an objective and reproducible manner. It consists of 4 grades where the severity of the complication increases with each grade. Grade 1 is any deviation from the normal post-operative course not requiring surgical, endoscopic or radiological intervention while grade 2 includes complications requiring drug treatments, such as blood transfusion and total parenteral nutrition. Grade 3 refers to complications requiring surgical, endoscopic or radiological intervention and grade 4 encompasses life-threatening complications which require intensive care.

This study was carried out under the approval of the Centralised Institutional Review Board of the Singapore Health Services (CIRB Reference No. 2015/2652).

### Statistical methods

Continuous variables were summarised using median and range as the data does not follow a normal distribution, and categorical variables by number and percentage of patients in each category. EORTC QLQ-C30 scores were summarised using mean, standard deviation, median and interquartile range. As the EORTC QLQ-C30 scores were not normally distributed, non-parametric tests were used. The Wilcoxon rank-sum test, with adjustment for ties, was used to test for differences in scores between different variables, namely age, sex, race, number of organs resected, recurrence and presence of post-operative complications. In addition, the Wilcoxon rank-sum test was used to evaluate for differences in scores across time. Where there were more than 2 groups, the Kruskal–Wallis rank test was implemented.

As the only data available from the reference group of NCCS patients were the mean and standard deviation of the scores, normality of the data had to be assumed and the 2-sample *t*-test was used to compare the scores obtained in the present study to that of the reference group. A 2-sided *p*-value of less than 0.05 was taken as statistically significant.

All analyses were performed using GraphPad Prism Version 7.04 (GraphPad Software, La Jolla, CA, USA).

## Results

**Fig. 1:**
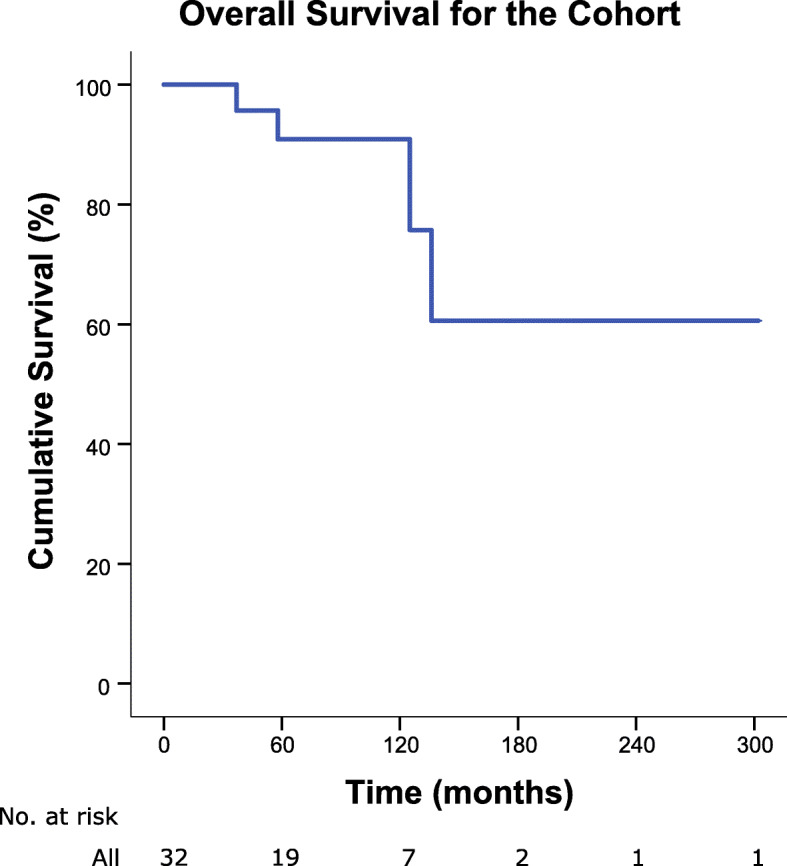
Overall survival outcome of patients

The characteristics of the patients are shown in Table 1. The median age was 59 years old (range, 35–84) with approximately equal numbers of both sexes. Majority of the patients were Chinese (87.5%), consistent with the racial distribution in the country and the most common histological subtype was liposarcoma (87.5%). The median number of organs resected was 1 (range, 0–4). The type of organs resected included adrenal, renal, gallbladder, spleen, pancreas and bowel. Twenty-one patients (65.6%) had a recurrence by the time of questionnaire administration and none had metastatic disease. All enrolled patients had completed their treatment, were disease-free, and under surveillance at the time of survey. The overall survival outcome of the included patients is shown in Fig. 1. 68.7% of the patients did not experience post-operative complications, with more than half of the remaining patients having grade 1–2 complications. 56.3% of the patients participated in the questionnaire when they were up to 2 years post-surgery, with the remaining being more than 2 years post- surgery.

**Table 1:** Summary of Patient Characteristics

Characteristic	Number	Percentage (%)
Total	32	100
Age (at diagnosis)		
Median (range)	59 (35–84)	
≤ 59 years	16	50.0
> 59 years	16	50.0
Sex		
Female	15	46.9
Male	17	53.1
Race		
Chinese	28	87.5
Indian	0	0
Others	4	12.5
Number of Organs Resected		
0	5	15.6
1–2	26	81.3
3–4	1	3.1
Histological Subtype		
Liposarcoma	28	87.5
Leiomyosarcoma	2	6.3
Fibromyxoid sarcoma	1	3.1
Synovial sarcoma	1	3.1
Adjuvant Therapy		
Radiotherapy	8	25.0
Chemotherapy	2	6.3
Recurrence (at time of questionnaire)		
No	11	34.4
Yes	21	65.6
Post-operative Complication		
None	22	68.7
Class I	1	3.1
Class II	6	18.8
Class III	3	9.4
Class IV	0	0
Time from initial surgery to questionnaire (years)		
< 0.5 years	6	18.8
0.5 to 1 year	6	18.8
1 to 2 years	5	15.6
> 2 years	15	46.8

### Summary of EORTC scores

A summary of the EORTC scores in the various functional domains and symptom scales is shown in Table 2. The mean global health score was 79.9 (SD = 18.6) with the highest functioning score in the cognitive domain at 93.6 (SD = 10.0) compared to social functioning at 84.9 (SD = 29.1). Fatigue and financial difficulties have the highest mean scores at 18.3 (SD = 23.7) and 13.7 (SD = 25.3) respectively while nausea and vomiting were the lowest at 2.6 (SD = 10.5).

**Table 2:** Summary of EORTC QLQ-C30 Scores

	N	Mean	Standard Deviation	Median	Minimum	Maximum	Interquartile range
Global health	32	79.9	18.6	79.2	33.3	100	33.3
Functional scales							
Physical functioning	32	87.3	20.9	93.3	20	100	10.8
Role functioning	32	91.7	19.9	100	0	100	16.7
Emotional functioning	32	87.2	21.2	100	0	100	18.8
Cognitive functioning	32	93.6	10.0	100	0	100	16.7
Social functioning	32	84.9	29.1	100	0	100	33.4
Symptom scales							
Fatigue	32	18.3	23.7	0	0	88.9	33.3
Nausea and vomiting	32	2.6	10.5	0	0	50	0
Pain	32	8.6	16.3	0	0	66.6	13.5
Dyspnoea	32	8.3	16.9	0	0	66.6	0
Insomnia	32	10.1	22.8	0	0	66.6	0
Appetite loss	32	6.2	17.8	0	0	66.6	0
Constipation	32	5.2	14.9	0	0	66.6	0
Diarrhoea	32	8.1	22.1	0	0	100	0
Financial difficulties	32	13.7	25.3	0	0	100	27.1

### Comparison of EORTC scores across variables

We compared the scores among different variables, namely age, sex, race, number of organs resected, recurrence and presence of post-operative complications (Table 3 and Supplementary Table 1). Younger patients had a significantly higher overall mean global health score of 88.2 points compared to 74.5 points in older patients (*p*-value = 0.040). Additionally, younger patients had significantly better physical (*p*-value = 0.030) and role functioning (*p*-value = 0.020). They also reported less fatigue compared to those who were older (*p*-value = 0.036). Female patients had higher global health scores (*p*-value = 0.014), physical (*p*-value = 0.016), emotional (*p*-value = 0.041) and social functioning (*p*-value = 0.009) while male patients experienced more fatigue (*p*-value = 0.003) and pain (*p*-value = 0.028) symptoms. Additionally, males reported more financial difficulties (*p*-value = 0.044). Patients who did not experience recurrence expressed better cognitive functioning than those who recurred (*p*-value = 0.044). No differences were observed across the various scales when we compared race, number of organs resected and post-operative complications.

**Table 3:** Comparison of EORTC Scores across Variables

	Age			Sex		Race		Organs Resected			Recurrence		Post-operative Complication			
Global health		**≤59**	**> 59**	**Female**	**Male**	**Chinese**	**Others**	**0**	**1–2**	**3–4**	**No**	**Yes**	**None**	**Class I**	**Class II**	**Class III**
	**Mean**	88.2	74.5	90.0	73.7	79.2	97.5	72.4	82.0	–	85.1	79.4	84.1	–	78.6	71.8
	***p*-value**	0.040		0.014		0.092		0.439			0.436		0.237			
Functioning Scales																
Physical functioning	**Mean**	95.1	79.4	96.4	79.1	85.8	96.7	69.3	90.1	–	92.3	84.6	88.0	–	89.2	77.8
	***p*-value**	0.030		0.016		0.300		0.432			0.330		0.755			
Role functioning	**Mean**	97.9	81.2	95.5	84.3	88.0	100	90.0	90.0	–	97.0	85.7	93.9	–	75.0	88.9
	***p*-value**	0.020		0.126		0.287		0.947			0.145		0.076			
Emotional functioning	**Mean**	90.0	79.2	92.5	77.6	83.2	93.8	82.9	84.7	–	91.3	81.0	86.5	–	78.4	83.3
	***p*-value**	0.145		0.041		0.354		0.411			0.191		0.455			
Cognitive functioning	**Mean**	96.1	91.1	95.5	91.9	92.7	100	90.8	93.7	–	98.5	91.1	93.0	–	94.4	94.4
	***p*-value**	0.165		0.311		0.176		0.411			0.044		0.607			
Social functioning	**Mean**	87.5	91.7	96.7	70.6	80.3	100	73.3	84.7	–	93.9	77.0	82.6	–	83.3	77.8
	***p*-value**	0.371		0.009		0.213		0.858			0.120		0.948			
Symptom scales																
Fatigue	**Mean**	10.8	27.7	6.7	30.4	22.0	0	38.9	17.8	–	15.1	21.4	15.4	–	25.9	29.6
	***p*-value**	0.036		0.003		0.076		0.433			0.477		0.167			
Nausea and vomiting	**Mean**	0	5.2	2.2	2.9	3.0	0	0	3.3	–	3.0	2.4	1.5	–	8.3	0
	***p*-value**	0.163		0.849		0.603		0.447			0.871		0.391			
Pain	**Mean**	3.6	13.5	1.9	14.4	9.8	0	29.1	5.2	–	4.1	10.9	5.7	–	13.9	22.2
	***p*-value**	0.086		0.028		0.266		0.966			0.272		0.136			
Dyspnoea	**Mean**	4.2	12.5	2.2	13.7	9.5	0	26.6	5.3	–	6.1	9.5	4.5	–	11.1	22.2
	***p*-value**	0.168		0.054		0.300		0.357			0.591		0.059			
Insomnia	**Mean**	4.2	16.1	6.1	13.7	11.6	0	26.6	6.7	–	8.3	11.1	11.7	–	0	22.2
	***p*-value**	0.141		0.356		0.351		0.509			0.750		0.569			
Appetite loss	**Mean**	4.2	8.3	6.7	5.9	7.1	0	6.7	6.7	–	3.0	7.9	7.6	–	0	11.1
	***p*-value**	0.518		0.904		0.463		0.280			0.469		0.542			
Constipation	**Mean**	4.2	6.2	2.2	7.8	4.8	8.3	0	6.7	–	3.0	6.3	7.6	–	0	0
	***p*-value**	0.700		0.295		0.662		0.086			0.559		0.188			
Diarrhoea	**Mean**	3.6	12.5	6.7	9.3	9.2	0	25.0	5.3	–	3.0	10.7	7.2	–	0	33.3
	***p*-value**	0.265		0.741		0.445		0.261			0.360		0.745			
Financial difficulties	**Mean**	12.0	14.6	3.9	21.6	14.3	6.3	40.0	9.0	–	9.1	15.5	8.7	–	11.1	44.4
	***p*-value**	0.774		0.044		0.557		0.593			0.502		0.128			

### Comparison of EORTC scores across time

We then grouped patients into two groups according to the time they were recruited into the study. The first group comprised patients who undertook the questionnaire when they were up to 2 years post-surgery, while the second group was made up of patients who were more than 2 years post-surgery (Table 4). The rationale for choosing 2 years post-surgery as a cut-off is based on the QOL trend seen from our data where scores generally rose again after 2 years. In particular, from the trend of our data, global health, physical functioning, role functioning, emotional functioning, cognitive functioning and social functioning scores were generally lower in the 6 months to 1 year and 1 to 2 years groups, however increased again in the group more than 2 years after. These differences trended towards significance for global health and social functioning. Due to the small sample size, the comparison of EORTC scores was not adjusted for differences in baseline characteristics.

**Table 4:** EORTC QLQ-C30 scores by time from surgery to questionnaire

Time to questionnaire	≤2 years (*N* = 17) Median	> 2 years (*N* = 15) Median	*p*-Value
Global health	71.4	100	0.077
Functional Scales			
Physical functioning	93.3	100	0.121
Role functioning	100	100	0.067
Emotional functioning	83.3	91.7	0.293
Cognitive functioning	100	100	0.386
Social functioning	83.3	100	0.054
Symptom Scales			
Fatigue	33.3	0	0.173
Nausea and vomiting	0	0	0.447
Pain	0	0	0.775
Dyspnoea	0	0	0.826
Insomnia	0	0	0.693
Appetite loss	0	0	0.948
Constipation	0	0	0.203
Diarrhoea	0	0	0.383
Financial difficulties	0	16.7	0.879

Global health score (*p*-value = 0.077), physical (*p*-value = 0.121), emotional (*p*-value = 0.293) and social (*p*-value = 0.054) functioning scales revealed an increasing trend across time; however these did not reach statistical significance. Role and cognitive functioning scales had median scores of 100 at both time points. All functional scales except emotional functioning reached a median score of 100 when patients were more than 2 years post-surgery. Additionally, no significant differences were observed for all symptom scales.

### Comparison of EORTC scores with a local cohort of Cancer patients

In comparison to the reference group of cancer patients from NCCS, our group had higher global health (*p*-value < 0.001), and emotional functioning scores (*p*-value = 0.033) (Table 5). Our patients also expressed better physical and social functioning score although these did not reach statistical significance.

**Table 5:** Comparison of EORTC QLQ-C30 scores between sarcoma patients and a reference group of cancer patients

	Retroperitoneal sarcoma patients		Control patients		Difference (95% CI)	*p*-Value
	No.	Mean (SD)	No.	Mean (SD)		
Global health	32	79.9 (18.6)	379	66.6 (19.9)	13.3 (6.1 to 20.5)	< 0.001
Physical functioning	32	87.3 (20.9)	379	84.8 (15.3)	2.5 (−3.2 to 8.2)	0.390
Emotional functioning	32	87.2 (21.2)	379	79.3 (20.0)	7.9 (0.6 to 15.2)	0.033
Social functioning	32	84.9 (29.1)	377	77.1 (25.6)	7.8 (−1.6 to 17.2)	0.102

## Discussion

Understanding QOL is critical for appropriately addressing a patient’s needs and treatment options in the local cultural context [12]. The shift from extending survival to delaying deterioration in patient-reported symptom, function and QOL is critical and an important goal of treatment. A better understanding of health-related QOL throughout the course of treatment as measured with appropriate patient-reported outcomes help to guide collaborative decision-making with patients. However, there is limited literature evidence on the QOL of patients with retroperitoneal sarcoma, particularly in an Asian population. To date, there is only one published QOL study of patients with retroperitoneal sarcoma in an Asian cohort consisting of 10 patients [13]. The study reported that tumour resection with preservation of important organs may improve patients’ quality of postoperative life and survival. With limited literature in this area, our study would be one of the first to pave the way for a prospective QOL in patients with retroperitoneal sarcoma.

Our study revealed significant differences in global health and various functioning scale scores amongst patients with retroperitoneal sarcoma, in terms of age and sex. This does not come surprising as these factors have been demonstrated in various studies to be affected following surgery. For example, Paredas et al. [14] reported that elderly cancer patients felt more socially isolated post-surgery, which was attributed to a smaller social circle and a change in relationship between patients and their partners as well as family members. Multiple reports have also shown that female patients are more receptive in seeking psychosocial support compared to males [15–17]. In addition, male patients may not be open to receiving emotional help due to cultural pressures of presenting a tough image. Instead, they prefer to manage their illnesses individually and conceal their emotions from family members [18, 19]. Nevertheless, interventions such as cognitive-behavioural, mindfulness, or family and social support to improve the QOL may be helpful [20, 21]. Our study did not show any difference in QOL by race. Notably, majority of our patients were Chinese with only 4 patients from other races. Hence, our study has shown that demographic variables may be predictors of QOL in patients with retroperitoneal sarcoma following surgery.

Due to the retrospective cross-sectional nature of our study, we were unable to obtain longitudinal data. However, our study revealed that patients who were less than 2 years post-surgery reported lower median global health and functional scale scores compared to those more than 2 years. There were no significant differences seen in symptom scores between both groups. We postulate that the lower global health and functional scores observed was due to disease recurrence during the first 2 years. Twenty-one patients (65.6%) in our study had a local recurrence with 38.1% having a recurrence within 2 years of initial surgery. Likewise, a study which evaluated the QOL in patients with retroperitoneal sarcoma treated with pre-operative radiotherapy and surgery reported that patients who survived and were free of recurrence at 36 months or more had significantly better QOL than at diagnosis [22]. Moreover, the association between disease recurrence and reduced QOL has been previously described in other malignancies [23, 24]. Hence, disease recurrence and possibly the treatment toxicities could account for the poorer scores in the first 2 years. Moreover, symptoms such as pain, vomiting and diarrhoea, and dyspnoea, have been adversely associated with QOL recovery in patients with retroperitoneal sarcoma [25, 26]. Nevertheless, the scores improved in the group which was more than 2 years post-surgery, suggesting that patients with retroperitoneal sarcoma need to be followed up for at least 2 years following surgery to gain insight into the impact of surgical intervention on patient outcomes. Additionally, we compared our results to a cohort of disease-free cancer patients who were on follow up in NCCS [11]. We presumed our patients’ QOL returned to baseline should they achieve comparable results to that of our reference group. Interestingly, our patients exhibited better scores than those from the reference cohort in all categories, particularly global health and emotional functioning scale. These results are encouraging as the reference cohort consisted of a subset of cancer patients in relatively good state of health where they were free of disease with ECOG status 0 or 1 and were not on active treatment. Extrapolating these results, patients with retroperitoneal sarcoma exhibited a temporal increasing trend of QOL scores post-surgery and returned to baseline. Similar results have also been reported in other malignancies such as peritoneal carcinomatosis and oral cancers [23, 24, 27, 28].

Our study has several limitations, including the retrospective nature, small sample size and different follow-up time points of included patients. As a retrospective study, this series is limited by the bias inherent with this methodology. As a significant number of patients had passed on due to the disease, a small sample size was used which might create a potential bias and may not be fully representative of the larger population. Accepting an alpha risk of 0.05 in a two-sided test, a sample size of 32 patients in our study would give a power of 44%. Our study population may also be inherently biased as the patients who are selected are only those who have survived and are on follow-up. Furthermore, we are looking at QOL scores from different patients at one point in time, hence direct comparisons across different groups have to be interpreted with caution. For instance, patients who have recurred are likely to be undergoing treatment and experiencing treatment-related morbidity, which could translate to poorer QOL. Finally, this study did not explore other determinants of QOL including body image, future perspective and systemic therapy side effects as reported in other cancer studies examining QOL [29]. Despite these limitations, there is still value in examining QOL of patients with retroperitoneal sarcoma following curative surgery. This study is one of the first few to examine the QOL of patients with retroperitoneal sarcoma following curative surgery and the first study in an Asian population. In the ideal setting, a prospective QOL will be useful as many patients with retroperitoneal sarcoma are symptomatic by the time they present. Additionally, it will provide greater insight on possible changes in QOL following surgery. Therefore, the findings from this study reinforce and support the need for a prospective study examining pre- and post-operative QOL at 3, 6 and 12 months, which is currently underway in our institution.

## Conclusion

Our study has provided a means to understand the trends of various aspects of QOL at different time points following surgery in patients with retroperitoneal sarcoma. Despite the complexities involved in the curative surgery for these patients, we have shown that patients with retroperitoneal sarcoma can achieve better functioning scores when compared with other cancer patients who were free of disease, highlighting the possibility of achieving reasonable QOL outcomes despite multi-visceral surgical resection. This study has also shown that patients need to be followed up for at least 2 years following surgery to evaluate their QOL. The evaluation and interpretation of results of similar studies bring the possibility for better treatment decisions in the future for patients with retroperitoneal sarcoma. Moving forward, this pilot study supports the need for further prospective studies to continue the evaluation of our patients’ QOL.

## Supplementary information

**Additional file 1 Supplementary Table 1**: Comparison of EORTC Scores across Variables

## Data Availability

The datasets used and/or analysed during the current study are available from the corresponding author on reasonable request.

## Abbreviations

QOL: Quality of life
NCCS: National cancer centre singapore
EORTC QLQ-C30: European organization for the research and treatment of cancer care quality of life questionnaire

## References

[1] Matthyssens LE, Creytens D, Ceelen WP (2015). Retroperitoneal Liposarcoma: current insights in diagnosis and treatment. Front Surg.

[2] Lee HS, Yu JI, Lim DH, Kim SJ (2016). Retroperitoneal liposarcoma: the role of adjuvant radiation therapy and the prognostic factors. Radiat Oncol J.

[3] Abbott AM, Habermann EB, Parsons HM, Tuttle T, Al-Refaie W (2012). Prognosis for primary retroperitoneal sarcoma survivors: a conditional survival analysis. Cancer.

[4] Cheung YB, Thumboo J, Goh C, Khoo KS, Che W, Wee J (2004). The equivalence and difference between the English and Chinese versions of two major, cancer-specific, health-related quality-of-life questionnaires. Cancer.

[5] Hogg H, Manas D, Lee D (2016). Surgical outcome and patterns of recurrence for retroperitoneal sarcoma at a single Centre. Ann R Coll Surg Engl.

[6] Wiegering A, Isbert C, Dietz UA, Kunzmann V, Ackermann S, Kerscher A, Maeder U, Flentje M, Schlegel N, Reibetanz J, Germer CT, Klein I. Multimodal therapy in treatment of rectal cancer is associated with improved survival and reduced local recurrence – a retrospective analysis over two decades. BMC Cancer. 2014;6;14:816. 10.1186/1471-2407-14-816 PMC4236459 25376382

[7] Wang Y, Shen L, Lu M, Ji Z, Zhang XT. Multimodality treatment including triplet regimen as first-line chemotherapy may improve prognosis of serum AFP-elevated gastric cancer with liver metastasis. Gastroenterol Res Pract. 2017;5080361. 10.1155/2017/5080361 PMC5757133 29434637

[8] Leffers N, Daemen T, van der Zee AG, Nijman HW (2009). Multimodality treatment warranted for ovarian cancer: immunotherapy, a prerequisite to improve prognosis for this vicious disease. Immunotherapy.

[9] Marventano S, Forjaz MJ, Grosso G (2013). Health related quality of life in colorectal cancer patients: state of the art. BMC Surg.

[10] Polanski J, Jankowska-Polanska B, Rosinczuk J, Chabowski M, Szymanska-Chabowska A (2016). Quality of life of patients with lung cancer. OncoTargets Ther.

[11] Cheung YB, Thumboo J, Goh C, Khoo KS, Che W, Wee J (2004). The equivalence and difference between the English and Chinese versions of two major, cancer-specific, health-related quality-of-life questionnaires. Cancer.

[12] Tran BX, Vu GT, Ha GH, et al. Global Mapping of Interventions to Improve the Quality of Life of People Living with HIV/AIDS: Implications for Priority Settings. AIDS Rev. 2020;12:1–15. 10.24875/AIDSRev.20000135 33105468

[13] Ikeguchi M, Urushibara S, Shimoda R, Saito H, Wakatsuki T (2014). Surgical treatment of retroperitoneal Liposarcoma. Yonago Acta Med.

[14] Paredas T, Pereira M, Moreira H, Simões MR, Canavarro MC. Quality of life of sarcoma patients from diagnosis to treatments: predictors and longitudinal trajectories. Eur J Oncol Nurs. 2011;5:492–9. 10.1016/j.ejon.2011.01.001 21306951

[15] Jones RL, Cesne AL (2018). Quality of life and patients’ expectations in soft tissue sarcoma. Future Oncol.

[16] Ussher J, Kirsten L, Butow P, Sandoval M (2006). What do cancer support groups provide which other supportive relationships do not? The experience of peer support groups for people with cancer. Soc Sci Med.

[17] Osoba D, Rodrigues G, Myles J (1998). Interpreting the significance of changes in health-related quality-of-life scores. J Clin Oncol.

[18] Zenz M, Zenz T, Tryba M, Strumpf M. Severe undertreatment of cancer pain: a 3-year survey of the German situation. J Pain Symptom Manage. 10(3):187–91. 10.1016/0885-3924(94)00122-2 7629412

[19] Livhits MJ, Yeh MW (2017). Multimodality treatment with surgery, external-beam radiation, and chemotherapy improves survival for selected patients with anaplastic thyroid cancer. Clin Thyroidol.

[20] Tran BX, Ha GH, Nguyen DN (2020). Global mapping of interventions to improve quality of life of patients with depression during 1990-2018. Qual Life Res.

[21] Tran BX, Harijanto C, Vu GT (2020). Global mapping of interventions to improve quality of life using mind-body therapies during 1990-2018. Complement Ther Med.

[22] Wong P, Kassam Z, Springer AN, Gladdy R, Chung P, Ringash J, Catton C (2017). Long-term quality of life of retroperitoneal sarcoma patients treated with pre-operative radiotherapy and surgery. Cureus.

[23] Zieren HU, Jacobi CA, Zieren J, Muller JM (1996). Quality of life following resection of oesophageal carcinoma. Br J Surg.

[24] Camilleri-Brennan J, Steele RJC (2001). The impact of recurrent rectal cancer on quality of life. Eur J Surg Oncol.

[25] Gough NJ, Smith C, Ross JR, Riley J, Judson I. Symptom burden, survival and palliative care in advanced soft tissue sarcoma. Sarcoma. 2011;325189. 10.1155/2011/325189 PMC3236373 22190862

[26] Kuo PY, Yen JTC, Parker GM, et al. The prevalence of pain in patients attending sarcoma outpatient clinics. Sarcoma. 2011;813483. 10.1155/2011/813483 PMC3103993 21647362

[27] Chia SC, Tan WJ, Wong JFS (2014). Quality of life in patients with peritoneal surface malignancies after cytoreductive surgery and hyperthermic intraperitoneal chemotherapy. Eur J Surg Oncol.

[28] Yan Y-B, Meng L, Liu Z-Q (2017). Quality of life in long-term oral cancer survivors: an 8-year prospective study in China. Oral Surg Oral Med Oral Pathol Oral Radiol.

[29] Ng ET, Ang RZ, Tran BX (2019). Comparing quality of life in breast Cancer patients who underwent mastectomy versus breast-conserving surgery: a meta-analysis. Int J Environ Res Public Health.

