# Supplementary material for: Retrospective quality of life study in patients with retroperitoneal sarcoma in an Asian population
Source: Health Qual Life Outcomes. 2020 Aug 6;18:270. doi: 10.1186/s12955-020-01491-0 (PMC7409714; doi:10.1186/s12955-020-01491-0)
Supplement: Supplementary file 1 — Additional file 1 Supplementary Table 1: Comparison of EORTC Scores across Variables [file 12955_2020_1491_MOESM1_ESM.docx]

**Title:** Retrospective Quality of Life Study in Patients with Retroperitoneal Sarcoma in an Asian Population

**Authors:** Hui Jun Lim^1^*, Chin-Ann Johnny Ong^1,2,3,4^*, Thakshayeni Skanthakumar^1^, Lisa Yuen Hong Mak^1^, Seettha Devi Wasudevan^1^, Joey Wee-Shan Tan^1,3^, Claramae Shulyn Chia^1,2^, Grace Hwei Ching Tan^1,2^, Melissa Ching Ching Teo^1,2^**

^1^Department of Sarcoma, Peritoneal and Rare Tumours (SPRinT), Division of Surgery and Surgical Oncology, National Cancer Centre Singapore, 11 Hospital Crescent, S169610

^2^Duke-NUS Medical School, 8 College Road, S169857

^3^Laboratory of Applied Human Genetics, Division of Medical Sciences, National Cancer Centre Singapore, 11 Hospital Crescent, S169610

^4^Institute of Molecular and Cell Biology, A*STAR Research Entities, 61 Biopolis Drive, S138673

* Equal contribution

** Corresponding author

Correspondence to:

Adj Professor Melissa Ching Ching Teo

Department of Sarcoma, Peritoneal and Rare Tumours (SPRinT), Division of Surgery and Surgical Oncology

National Cancer Centre Singapore

11 Hospital Crescent

Singapore 169610

melissa.teo.c.c@singhealth.com.sg

**Supplementary Table 1: Comparison of EORTC Scores across Variables**

|  | Age | | | Gender | | | Race | | Organs Resected | | | Recurrence | | Post-operative Complication | | | |
| --- | --- | --- | --- | --- | --- | --- | --- | --- | --- | --- | --- | --- | --- | --- | --- | --- | --- |
| Global health |  | **≤59** | **>59** | **Female** | **Male** | | **Chinese** | **Others** | **0** | **1-2** | **3-4** | **No** | **Yes** | **None** | **Class I** | **Class II** | **Class III** |
|  | **Mean** | 88.2 | 74.5 | 90.0 | 73.7 | | 79.2 | 97.5 | 72.4 | 82.0 | - | 85.1 | 79.4 | 84.1 | - | 78.6 | 71.8 |
|  | **Median** | 100 | 71.4 | 100 | 71.4 | | 73.2 | 100 | 71.4 | 83.3 | - | 100 | 75.0 | 96.5 | - | 77.4 | 66.7 |
|  | **SD** | 15.8 | 20.1 | 13.4 | 20.5 | | 19.4 | 7.2 | 28.7 | 17.3 | - | 21.9 | 17.7 | 19.5 | - | 19.8 | 17.9 |
|  | **p-value** | 0.040 | | 0.014 | | | 0.092 | | 0.439 | | | 0.436 | | 0.237 | | | |
| Functioning Scales | | | | | | | | | | | | | | | | | |
| Physical functioning | **Mean** | 95.1 | 79.4 | 96.4 | 79.1 | | 85.8 | 96.7 | 69.3 | 90.1 | - | 92.3 | 84.6 | 88.0 | - | 89.2 | 77.8 |
|  | **Median** | 100 | 93.3 | 100 | 93.3 | | 93.3 | 100 | 90.0 | 100 | - | 100 | 93.3 | 100 | - | 93.3 | 93.3 |
|  | **SD** | 7.4 | 26.6 | 6.0 | 25.7 | | 21.8 | 5.0 | 29.9 | 18.1 | - | 19.8 | 21.3 | 21.7 | - | 14.5 | 32.9 |
|  | **p-value** | 0.030 | | 0.016 | | | 0.300 | | 0.432 | | | 0.330 | | 0.755 | | | |
| Role functioning | **Mean** | 97.9 | 81.2 | 95.5 | 84.3 | | 88.0 | 100 | 90.0 | 90.0 | - | 97.0 | 85.7 | 93.9 | - | 75.0 | 88.9 |
|  | **Median** | 100 | 91.7 | 100 | 100 | | 100 | 100 | 100 | 100 | - | 100 | 100 | 100 | - | 91.7 | 100 |
|  | **SD** | 5.7 | 26.5 | 11.8 | 25.3 | | 21.7 | 0 | 14.9 | 22.1 | - | 10.1 | 23.7 | 12.1 | - | 39.1 | 19.3 |
|  | **p-value** | 0.020 | | 0.126 | | | 0.287 | | 0.947 | | | 0.145 | | 0.076 | | | |
| Emotional functioning | **Mean** | 90.0 | 79.2 | 92.5 | 77.6 | | 83.2 | 93.8 | 82.9 | 84.7 | - | 91.3 | 81.0 | 86.5 | - | 78.4 | 83.3 |
|  | **Median** | 92.7 | 83.3 | 100 | 83.3 | | 85.4 | 93.8 | 87.5 | 87.5 | - | 100 | 83.3 | 96.9 | - | 83.3 | 100 |
|  | **SD** | 12.6 | 26.0 | 9.3 | 25.6 | | 21.9 | 7.2 | 19.4 | 21.6 | - | 11.9 | 23.8 | 22.9 | - | 9.3 | 28.9 |
|  | **p-value** | 0.145 | | 0.041 | | | 0.354 | | 0.411 | | | 0.191 | | 0.455 | | | |
| Cognitive functioning | **Mean** | 96.1 | 91.1 | 95.5 | 91.9 | | 92.7 | 100 | 90.8 | 93.7 | - | 98.5 | 91.1 | 93.0 | - | 94.4 | 94.4 |
|  | **Median** | 100 | 95.6 | 100 | 100 | | 100 | 100 | 87.5 | 100 | - | 100 | 100 | 100 | - | 100 | 100 |
|  | **SD** | 9.3 | 10.3 | 9.9 | 10.1 | | 10.4 | 0 | 8.6 | 10.6 | - | 5.0 | 11.1 | 10.9 | - | 8.6 | 9.6 |
|  | **p-value** | 0.165 | | 0.311 | | | 0.176 | | 0.411 | | | 0.044 | | 0.607 | | | |
| Social functioning | **Mean** | 87.5 | 91.7 | 96.7 | 70.6 | | 80.3 | 100 | 73.3 | 84.7 | -- | 93.9 | 77.0 | 82.6 | - | 83.3 | 77.8 |
|  | **Median** | 100 | 91.7 | 100 | 66.6 | | 100 | 100 | 66.6 | 100 | - | 100 | 100 | 100 | - | 83.3 | 100 |
|  | **SD** | 29.5 | 29.1 | 9.4 | 35.1 | | 30.5 | 0 | 27.9 | 30.4 | - | 13.5 | 33.5 | 31.9 | - | 18.3 | 38.5 |
|  | **p-value** | 0.371 | | 0.009 | | | 0.213 | | 0.858 | | | 0.120 | | 0.948 | | | |
| Symptom scales | | | | | | | | | | | | | | | | | |
| Fatigue | **Mean** | 10.8 | 27.7 | 6.7 | 30.4 | | 22.0 | 0 | 38.9 | 17.8 | - | 15.1 | 21.4 | 15.4 | - | 25.9 | 29.6 |
|  | **Median** | 0 | 27.8 | 0 | 33.3 | | 19.5 | 0 | 44.5 | 83.3 | - | 0 | 22.2 | 0 | - | 27.8 | 33.3 |
|  | **SD** | 14.4 | 27.5 | 13.8 | 24.5 | | 23.6 | 0 | 29.4 | 22.2 | - | 22.9 | 23.7 | 24.8 | - | 15.2 | 28.0 |
|  | **p-value** | 0.036 | | 0.003 | | | 0.076 | | 0.433 | | | 0.477 | | 0.167 | | | |
| Nausea and vomiting | **Mean** | 0 | 5.2 | 2.2 | | 2.9 | 3.0 | 0 | 0 | 3.3 | - | 3.0 | 2.4 | 1.5 | - | 8.3 | 0 |
|  | **Median** | 0 | 0 | 0 | | 0 | 0 | 0 | 0 | 0 | - | 0 | 0 | 0 | - | 0 | 0 |
|  | **SD** | 0 | 14.5 | 8.6 | | 12.1 | 11.2 | 0 | 0 | 11.8 | - | 10.0 | 10.9 | 7.1 | - | 20.4 |  |
|  | **p-value** | 0.163 | | 0.849 | | | 0.603 | | 0.447 | | | 0.871 | | 0.391 | | | |
| Pain | **Mean** | 3.6 | 13.5 | 1.9 | | 14.4 | 9.8 | 0 | 29.1 | 5.2 | - | 4.1 | 10.9 | 5.7 | - | 13.9 | 22.2 |
|  | **Median** | 0 | 0 | 0 | | 0 | 0 | 0 | 16.6 | 0 | - | 0 | 0 | 0 | - | 8.3 | 0 |
|  | **SD** | 9.0 | 20.4 | 5.2 | | 20.3 | 17.1 | 0 | 27.9 | 10.3 | - | 7.2 | 19.2 | 10.6 | - | 19.5 | 38.5 |
|  | **p-value** | 0.086 | | 0.028 | | | 0.266 | | 0.966 | | | 0.272 | | 0.136 | | | |
| Dyspnoea | **Mean** | 4.2 | 12.5 | 2.2 | | 13.7 | 9.5 | 0 | 26.6 | 5.3 | - | 6.1 | 9.5 | 4.5 | - | 11.1 | 22.2 |
|  | **Median** | 0 | 0 | 0 | | 0 | 0 | 0 | 33.3 | 0 | - | 0 | 0 | 0 | - | 0 | 0 |
|  | **SD** | 11.4 | 20.6 | 8.6 | | 20.6 | 17.8 | 0 | 27.9 | 12.5 | - | 13.5 | 18.7 | 11.7 | - | 17.2 | 38.5 |
|  | **p-value** | 0.168 | | 0.054 | | | 0.300 | | 0.357 | | | 0.591 | | 0.059 | | | |
| Insomnia | **Mean** | 4.2 | 16.1 | 6.1 | | 13.7 | 11.6 | 0 | 26.6 | 6.7 | - | 8.3 | 11.1 | 11.7 | - | 0 | 22.2 |
|  | **Median** | 0 | 0 | 0 | | 0 | 0 | 0 | 0 | 0 | - | 0 | 0 | 0 | - | 0 | 0 |
|  | **SD** | 16.7 | 26.9 | 17.9 | | 26.5 | 24.1 | 0 | 36.5 | 19.2 | - | 20.7 | 24.3 | 23.9 | - | 0 | 38.5 |
|  | **p-value** | 0.141 | | 0.356 | | | 0.351 | | 0.509 | | | 0.750 | | 0.569 | | | |
| Appetite loss | **Mean** | 4.2 | 8.3 | 6.7 | | 5.9 | 7.1 | 0 | 6.7 | 6.7 | - | 3.0 | 7.9 | 7.6 | - | 0 | 11.1 |
|  | **Median** | 0 | 0 | 0 | | 0 | 0 | 0 | 0 | 0 | - | 0 | 0 | 0 | - | 0 | 0 |
|  | **SD** | 16.7 | 19.2 | 18.7 | | 17.6 | 18.9 | 0 | 14.9 | 19.2 | - | 10.0 | 20.8 | 20.4 | - | 0 | 19.2 |
|  | **p-value** | 0.518 | | 0.904 | | | 0.463 | | 0.280 | | | 0.469 | | 0.542 | | | |
| Constipation | **Mean** | 4.2 | 6.2 | 2.2 | | 7.8 | 4.8 | 8.3 | 0 | 6.7 | - | 3.0 | 6.3 | 7.6 | - | 0 | 0 |
|  | **Median** | 0 | 0 | 0 | | 0 | 0 | 0 | 0 | 0 | - | 0 | 0 | 0 | - | 0 | 0 |
|  | **SD** | 11.4 | 18.1 | 8.6 | | 18.7 | 14.9 | 16.7 | 0 | 16.7 | - | 10.0 | 17.0 | 17.6 | - | 0 | 0 |
|  | **p-value** | 0.700 | | 0.295 | | | 0.662 | | 0.086 | | | 0.559 | | 0.188 | | | |
| Diarrhoea | **Mean** | 3.6 | 12.5 | 6.7 | | 9.3 | 9.2 | 0 | 25.0 | 5.3 | - | 3.0 | 10.7 | 7.2 | - | 0 | 33.3 |
|  | **Median** | 0 | 0 | 0 | | 0 | 0 | 0 | 0 | 0 | - | 0 | 0 | 0 | - | 0 | 0 |
|  | **SD** | 10.1 | 29.5 | 18.7 | | 25.3 | 23.5 | 0 | 43.3 | 15.7 | - | 10.0 | 26.2 | 17.1 | - | 0 | 57.7 |
|  | **p-value** | 0.265 | | 0.741 | | | 0.445 | | 0.261 | | | 0.360 | | 0.745 | | | |
| Financial difficulties | **Mean** | 12.0 | 14.6 | 3.9 | | 21.6 | 14.3 | 6.3 | 40.0 | 9.0 | - | 9.1 | 15.5 | 8.7 | - | 11.1 | 44.4 |
|  | **Median** | 0 | 0 | 0 | | 0 | 0 | 0 | 33.3 | 0 | - | 0 | 0 | 0 | - | 0 | 33.3 |
|  | **SD** | 20.2 | 29.7 | 10.4 | | 31.0 | 26.3 | 12.5 | 43.5 | 17.6 | - | 21.5 | 26.9 | 20.6 | - | 17.2 | 50.9 |
|  | **p-value** | 0.774 | | 0.044 | | | 0.557 | | 0.593 | | | 0.502 | | 0.128 | | | |
